# Gamma-tocotrienol and hydroxy-chavicol synergistically inhibits growth and induces apoptosis of human glioma cells

**DOI:** 10.1186/1472-6882-14-213

**Published:** 2014-07-01

**Authors:** Amirah Abdul Rahman, A Rahman A Jamal, Roslan Harun, Norfilza Mohd Mokhtar, Wan Zurinah Wan Ngah

**Affiliations:** 1 UKM Medical Molecular Biology Institute (UMBI), UKM Medical Center, Jalan Ya’acob, Bandar Tun Razak, Cheras, Kuala Lumpur 56000, Malaysia; 2 Department of Biochemistry, Faculty of Medicine, Universiti Kebangsaan Malaysia, Jalan Raja Muda Abdul Aziz, Kuala Lumpur 50300, Malaysia

**Keywords:** Gamma-tocotrienol, Hydroxy-chavicol, Synergism, Cytotoxicity, Apoptosis, Glioma

## Abstract

**Background:**

Gamma-tocotrienol (GTT), an isomer of vitamin E and hydroxy-chavicol (HC), a major bioactive compound in *Piper betle,* has been reported to possess anti-carcinogenic properties by modulating different cellular signaling events. One possible strategy to overcome multi-drug resistance and high toxic doses of treatment is by applying combinational therapy especially using natural bioactives in cancer treatment.

**Methods:**

In this study, we investigated the interaction of GTT and HC and its mode of cell death on glioma cell lines. GTT or HC alone and in combination were tested for cytotoxicity on glioma cell lines 1321N1 (Grade II), SW1783 (Grade III) and LN18 (Grade IV) by [3-(4,5-dimethylthiazol-2- yl)-5-(3-carboxymethoxy-phenyl)-2-(4-sulfophenyl)- 2H- tetrazolium, inner salt] MTS assay. The interactions of each combination were evaluated by using the combination index (CI) obtained from an isobologram.

**Results:**

Individually, GTT or HC displayed mild growth inhibitory effects against glioma cancer cell lines at concentration values ranging from 42–100 μg/ml and 75–119 μg/ml respectively. However, the combination of sub-lethal doses of GTT + HC dramatically enhanced the inhibition of glioma cancer cell proliferation and exhibited a strong synergistic effect on 1321N1 with CI of 0.55, and CI = 0.54 for SW1783. While in LN18 cells, moderate synergistic interaction of GTT + HC was observed with CI value of 0.73. Exposure of grade II, III and IV cells to combined treatments for 24 hours led to increased apoptosis as determined by annexin-V FITC/PI staining and caspase-3 apoptosis assay, showing caspase-3 activation of 27%, 7.1% and 79% respectively.

**Conclusion:**

In conclusion, combined treatments with sub-effective doses of GTT and HC resulted in synergistic inhibition of cell proliferation through the induction of apoptosis of human glioma cells *in vitro*.

## Background

Despite aggressive therapy attempts, mortality from malignant astrocytic gliomas, the most common intrinsic brain tumors in adults, still remains unacceptably high partly due to chemo-resistance [1]. Patients with glioblastoma multiforme (GBM) WHO grade IV have a poor prognosis with a median overall survival of approximately 1 year and fewer than 5% of patients will survive 5 years [2]. For anaplastic astrocytoma WHO grade III and its low-grade counterpart, diffuse astrocytoma WHO grade II, the median overall patients’ survival is typically 3 to 5 years [3]. The use of alkylating substance such as temozolomide (TMZ) in treating cancer patients is limited due to resistance of tumor cells exhibiting increased activity of the DNA repair enzyme O^6^-alkylguanine-DNA alkyltransferase [4]. Thus, the development of alternative therapeutic strategies and to prevent recurrence is indicated.

Natural compounds with high effectiveness and fewer side effects are desirable as substitutes for chemical treatments which have various adverse effects. Alternative therapeutic approaches such as the use of non-cytotoxic dietary bioactives have potential for brain cancer because many of these natural compounds possess pleiotropic properties. To date, most mechanistic studies on chemopreventive agents have utilized a single dietary bioactive at fairly high concentrations which is unlikely to be achieved by food intake [5]. Thus, combination of bioactives is an alternative approach to uncover promising treatments either as an adjuvant therapy or in the prevention of recurrence, for cancer which are targeted and less toxic. Before entering the clinical trial phase which is very expensive, biomedical studies involving *in vitro* screening and quantification of synergy is an approach to generate fast, easy and robust data [6].

In this study, the concept of synergy interaction was tested by analyzing the efficacy of gamma-tocotrienol (GTT) and hydroxy-chavicol (HC) bioactives individually and in combination against glioma cancer cell lines. Tocotrienols possess more powerful anticancer, neuroprotective and cholesterol-lowering properties that are often not exhibited by tocopherols [7]. GTT, an isomer of the vitamin E family from palm oil has been reported to have anticancer activity and potent chemopreventive effects on tumor cells. The effects of GTT have been studied in colon and prostate cancer where GTT has been found to modulate multiple signaling pathways and induce apoptotic cell death [8]. Mechanisms involve modulation of various signaling pathways including apoptosis by caspase-8 activation and mitochondrial dependency, inhibition of cell proliferation, down-regulation of cyclins, reduction in the Pl3K/PDK-1/Akt signaling and NFκb activity and modulation of p53, Bax/Bcl2. Recently, Yap and colleagues reported the modulation of ID family proteins and mesenchymal markers in prostate and breast cancer cells in response to GTT [9].

The deep green heart-shaped leaves commonly referred to as “betel leaves” are traditionally consumed as a mouth freshener in Eastern Asia. Hydroxy-chavicol (4-allyl-catechol, 1-allyl-3,4-dihydroxybenzene, HC), a major phenolic compound in *Piper betle* leaves which is frequently found in a traditional Asian diet and remedies, has been shown to induce cell apoptosis by the induction of oxidative stress, glutathione (GSH) depletion and cell cycle deregulation [10]. HC also exert its antitumor effects by enhancing the immune response [11] and induce apoptosis by affecting the mitochondrial signaling pathway and modulating p53 [12]. Previous studies have suggested that HC exerts antioxidant, anti-inflammatory [13], anti-nitrosation, anti-mutagenic [14] and anti-carcinogenic properties against various mutagens and carcinogens [10].

Synergism can be acquired if the element of bioactive mixtures affect distinct targets or interact with one another to improve the solubility and enhance the bioavailability of one or several substances of the combined-compounds in combination [15]. In theory, combination of compounds can affect several targets, such as enzymes, substrates, metabolites, receptors, ion channels, DNA/RNA, monoclonal antibodies, signal cascades and physicochemical mechanisms [16]. Since these GTT and HC possess their own unique activities, we investigate the nature of interaction of these compounds by treating different grades of glioma cells to a sub-effective dose of combined GTT + HC, followed by the determination of cell proliferation and apoptosis by the presence of caspase-3 and annexin-V FITC/PI. We report the optimized combination ratio of GTT and HC compounds on grade II, III and IV glioma cells.

## Methods

### Reagents and chemicals

Gamma-tocotreinol (GTT) was purchased from Davos Life Science Pte Ltd. (Singapore) and hydroxy-chavicol (HC) from Hangzhou Imaginechem Co. Ltd. (Hangzhou, China). FITC Active Caspase-3 Apoptosis Kit and FITC Annexin V Apoptosis Detection Kit were purchased from BD Biosciences (USA). Other chemicals used were all of analytical grade.

### Cell line and culture condition

Human glioblatoma cell lines 1321N1 were purchased from the European Colection of Cell Culture (ECACC), while SW1783 and LN18 were obtained from American Type Culture Collection (ATCC) (Manassas, VA, USA). 1321N1 and LN18 were cultured in Dulbecco’s modified Eagle medium (DMEM) supplemented with penicillin, streptomycin, 10% fetal bovine serum (FBS) and 5% FBS respectively in a humidified incubator at 37°C in an atmosphere of 95% air and 5% CO_2_. SW1783 was maintained in Leibovitz, 10% FBS, in an atmosphere of 100% air. Medium was changed three times a week, and cells were passaged using accutase.

### Treatments with natural compounds

Stock solutions of GTT and HC were prepared in absolute ethanol and stored at −20°C. As vehicle, 0.1% of ethanol was added to control cells.

### Determination of cell viability

Viability of glioblastoma cancer cell lines treated with four phytochemical compounds and their combinations was assessed using CellTiter 96® Aqueous Non-Radioactive Cell Proliferation Assay (Promega, USA) as previously described. Briefly, cells were seeded in 96-well microtiter plates (Nunc) at 1.0 × 10^4^ per well. After 24 hour incubation, the medium was removed and the cells were treated with 100 μl medium containing various concentrations (50, 100, 150, 200 μg/ml) of GTT or HC compound alone for 24 hours. After 24 hour incubation, the medium was carefully removed, replaced with fresh medium, 20 μl of [3-(4,5-dimethylthiazol-2- yl)-5-(3-carboxymethoxy-phenyl)-2-(4-sulfophenyl)- 2H- tetrazolium, inner salt] (MTS) was added to each well and incubated at 37°C for 2 hours. The absorbance was measured at 490 nm in a VersaMax ELISA micro plate reader (Molecular Device, USA). The percentage of viable cells at each concentration was calculated by dividing the absorbance (A490) of treated cells by that of control cells. The half maximal inhibitory concentration (IC50) was determined from the cell viability (%) vs. concentrations graph. For bioactive compounds (GTT + HC) in combination, ½ or ¼ of IC50 of GTT were initially titrated to a range of concentrations (1, 10, 50, 100 μg/ml) of HC. All assays were performed in triplicates and repeated in three independent experiments.

### Active caspase-3 apoptosis assay

The presence of active caspase-3 using FITC Active Caspase-3 Apoptosis Kit. Cells were plated in 60 mm culture dish at a seeding density of 5 × 10^5^ cells/dish. GTT or HC was dissolved in ethanol, added to the culture media to the specified final concentration. As vehicle, 0.1% of ethanol was added to control cells. Camptothecin (CPT) was used as a positive control for apoptosis induction. After 24 h, cells were harvested and washed twice with PBS. Assays were performed as described in the manufacturer’s protocol. Briefly, cells were fixed in BD Cytofix/Cytoperm solution, incubated in ice for 20 min, washed with BD Perm/Wash buffer and FITC rabbit anti-active caspase-3 antibody was added and incubated for 30 min at room temperature. Fluorescence from a population of 1 × 10^5^ cells were detected using the BD FACSCanto^TM^ flow cytometer (Becton Dickenson, Mountain View, CA, USA) and CellQuest Pro (IVD) software (Becton Dickenson, Mountain View, CA, USA). Assays were performed in duplicates and repeated in three independent experiments.

### Annexin V-propidium iodide staining apoptosis assay

Apoptosis was determined using FITC Annexin V Apoptosis Detection Kit based on the membrane changes (phosphatidylserin based). Cells were plated in 60 mm culture dish at a seeding density of 5 × 10^5^ cells/dish. GTT or HC and GTT + HC in combination were dissolved in ethanol, added to the culture media to the specified final concentration. Vehicle was added alone to the culture medium serving as the untreated control. The subsequent procedures were carried out according to the instructions provided by the manufacturer. Briefly, after 24 h, cells were harvested, washed twice with PBS and resuspended in 1X binding buffer. Annexin-V FITC and propidium iodide (PI) were added and incubated for 15 min at room temperature (25°C) in the dark. Fluorescence from a population of 1 × 10^5^ cells were detected using the BD FACSCanto^TM^ flow cytometer (Becton Dickenson, Mountain View, CA, USA) and CellQuest Pro (IVD) software (Becton Dickenson, Mountain View, CA, USA). The assays were done in duplicates and repeated in three independent experiments.

### Statistical analysis

The level of interaction between the two bioactives were determined by isobologram analysis based on the Chou-Talalay method [17,18] where the output is represented as combination indexes (CI). The CI between two compounds A and B is:

$$\mathrm{CI}=\left(\frac{C_{A,X}}{IC_{X.A}}\right)+\left(\frac{C_{B,X}}{IC_{X.B}}\right)$$

Based on CI values, the extent of synergism/antagonism was determined. In brief, CI values between 0.9 and 0.85 suggest a moderate synergy, whereas those in the range of 0.7 to 0.3 are indicative of clear synergistic interactions between the drugs. CI values in the range of 0.9 to 1.10 suggest a near additive effect.

Statistical analysis among the various treatment groups in cell viability and apoptosis studies were performed by SPSS 16.0 software using two-tailed Student’s *t*-test and *P* < 0.05 were considered statistically significant. The data were expressed as mean ± standard deviation (SD).

## Results

### Effect of GTT, HC and bioactives in combination on the viability of glioma cells

**Figure 1:**
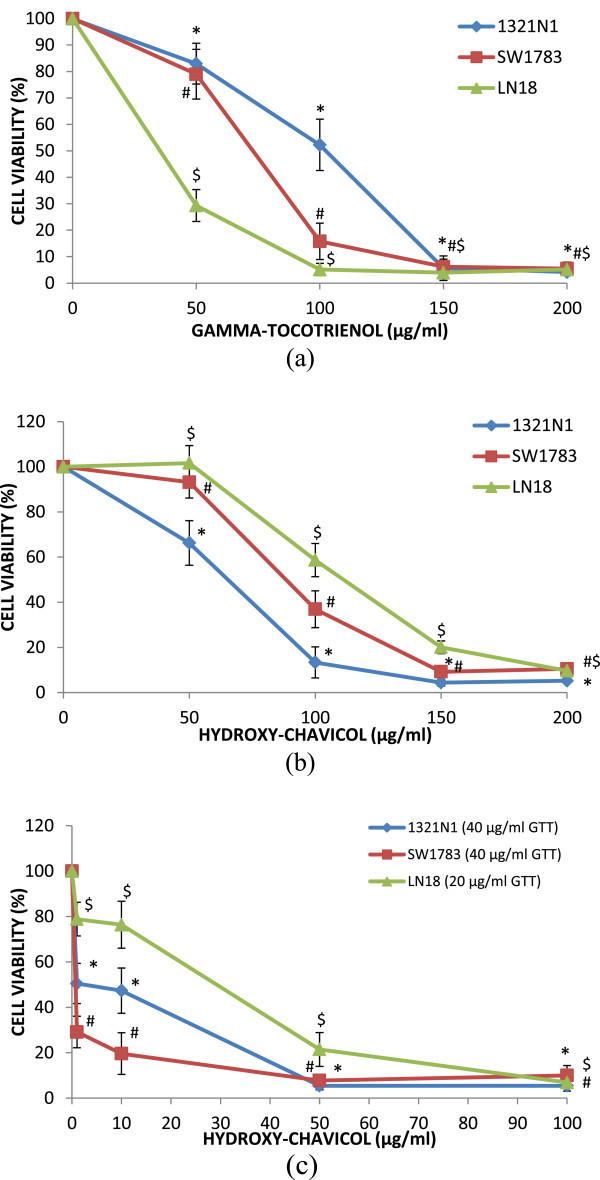
**Treatment of (a) gamma-tocotrienol (GTT), (b) hydroxyl-chavicol (HC) and (c) combination of GTT with HC; on 1321N1, SW1783 and LN18 for 24 h.** The cell survival test was determined by MTS assay. Data is presented as means ± SD, *n* = 9. *P < 0.05 compare to untreated 1321N1; # *P* < 0.05 compare to untreated SW1783; $*P* < 0.05 compare to untreated LN18.

Treatment with varying doses of GTT or HC on 1321N1, SW1783 and LN18 cells showed that cytotoxicity induced by GTT and HC was dose dependent with 90-95% inhibition achieved at maximum concentration of 200 μg/ml after 24 h of treatment (Figure 1(a) and (b)). Proliferation of 1321N1 cells decreased when treated with GTT resulting in a 50% reduction at 100 ± 10.22 μg/ml, while SW1783 showed a significant decrease in proliferation with 50% reduction at 79 ± 5.29 μg/ml, whereas the inhibitory concentration at 50% cell death (IC50) values of GTT for LN18 was 42 ± 2.52 μg/ml. The IC50 value of HC for 1321N1 was 75 ± 7.51 μg/ml, while SW1783 cell proliferation were 50% inhibited at IC50 of 95 ± 5.83 μg/ml, while 50% of LN18 cell proliferation were inhibited at 119 ± 7.77 μg/ml (Table 1). Grade IV, LN18 cells are more sensitive to GTT treatment alone compare to grade II 1321N1 and grade III SW1783 cell lines. However, grade II 1321N1 cells are more susceptible to HC treatment compared to other cell lines with a lower IC50 value.

**Table 1:** MTS cytotoxic effect of GTT and HC on human glioma cancer cells (1321N1, SW1783, LN18)

**Cell lines**	**Compound**	**IC50 value (μg/ml)**	**Viability (% cells)**^**a**^
Grade II	Gamma-tocotrienol (GTT)	100 ± 10.22	4.2 ± 1.34
1321N1	Hydroxy-chavicol (HC)	75 ± 7.51	5.2 ± 0.89
Grade III	Gamma-tocotrienol (GTT)	79 ± 5.29	5.5 ± 2.48
SW1783	Hydroxy-chavicol (HC)	95 ± 5.83	10.5 ± 2.04
Grade II	Gamma-tocotrienol (GTT)	42 ± 2.52	5.1 ± 1.82
LN18	Hydroxy-chavicol (HC)	119 ± 7.77	9.6 ± 1.66

^a^Percentage of cell viability after 24 h incubation of maximum concentration treatment (200 μg/ml) of each compound.

Viable cells (%) were expressed as the mean ± SD of three independent experiments.

**Figure 2:**
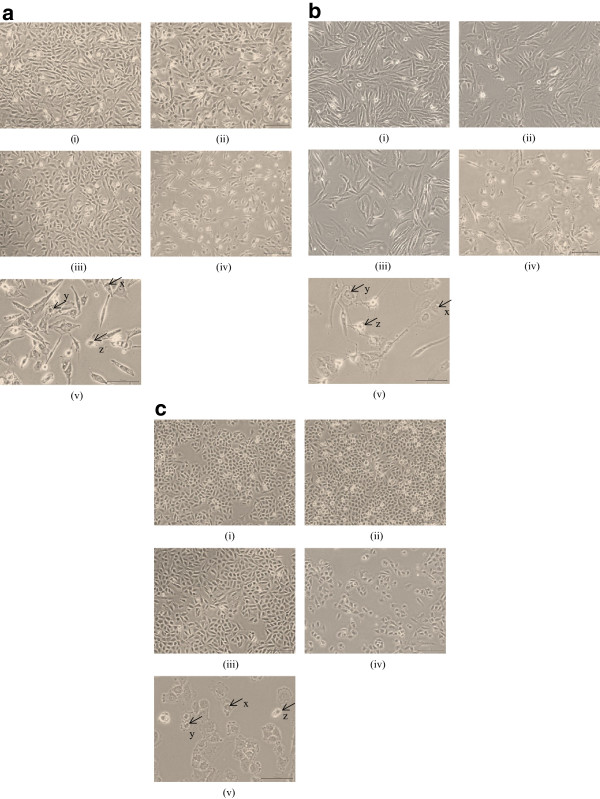
**Morphological changes of 1321N1, SW1783 and LN18 cells after treatment. a**. Morphological changes of 1321N1 cells after treatment with (i) vehicle untreated cells (ii) GTT 40 μg/ml, (iii) HC 12 μg/ml, (iv) 40 μg/ml of GTT + 12 μg/ml of HC, (v) scale 100 μm; 40 μg/ml of GTT + 12 μg/ml of HC; for 24 h. Arrow shows; x. chromatin margination (cresents), y. the collapse of nuclease and vacuolized cytoplasm, and z. cell shrinkage. **b**. Morphological changes of SW1783 cells after treatment with (i) vehicle untreated cells (ii) GTT 40 μg/ml, (iii) HC 2 μg/ml, (iv) 40 μg/ml of GTT + 2 μg/ml of HC, (v) scale 100 μm; 40 μg/ml of GTT + 2 μg/ml of HC; for 24 h. Arrow represents; x. collapse of nuclease, y. volume loss and chromatin clumping, and z. cell shrinkage. **c**. Morphological changes of LN18 cells after treatment with (i) vehicle untreated cells (ii) GTT 20 μg/ml, (iii) HC 29 μg/ml, (iv) 20 μg/ml of GTT + 29 μg/ml of HC, (v) scale 100 μm; 20 μg/ml of GTT + 29 μg/ml of HC; for 24 h. Arrow represents x. volume loss and chromatin clumping, y. collapse of nuclease, and z. cell shrinkage.

Combined GTT + HC significantly inhibited the growth of 1321N1, SW1783 and LN18 cells in a dose-dependent manner (Figure 1(c)), where the growth inhibition of cells affected by combined bioactives was found to be greater than either compound alone at lower doses, yielding combined IC50 values of 40 μg/ml of GTT + 12 μg/ml of HC in 1321N1 cells, 40 μg/ml of GTT + 2 μg/ml of HC in SW1783 cells, and 20 μg/ml of GTT + 29 μg/ml of HC in LN18 cell lines (Table 2). Moreover, combined GTT + HC were noted to induce apoptotic morphological changes such as membrane changes, condensation of chromatin, vacuoles in the cytoplasmic membrane and shrinkage of the cell size [19,20] in the glioma cells when treated with IC50 doses by microscopic examination (Figure 2a,b,c).

**Table 2:** The ratio of combined GTT and HC compounds at growth inhibition of 50% (IC50) on glioma cancer 1321N1, SW1783, LN18 cells and combination index (CI) for each combination

**Type of cell line**	**GTT:HC**	**IC50**^**a **^**[μg/ml]**	**GTT**^**b **^**[μg/ml]**	**HC**^**b **^**[μg/ml]**	**Combination Index**^**c **^**(CI)**
1321N1	10 : 3	12	100	75	0.55 ± 0.07
SW1783	20 : 1	2	79	95	0.54 ± 0.03
LN18	2 : 3	29	42	119	0.73 ± 0.03

^a^IC50 of combined compounds.

^b^IC50 of individual compound.

^c^CI < 1.0 indicates synergism; 0.9 < CI < 1.10, near additive; CI > 1.10 indicates antagonism.

The data are the average of three independent experiments.

### Analysis of interaction between combinations of compounds

**Figure 3:**
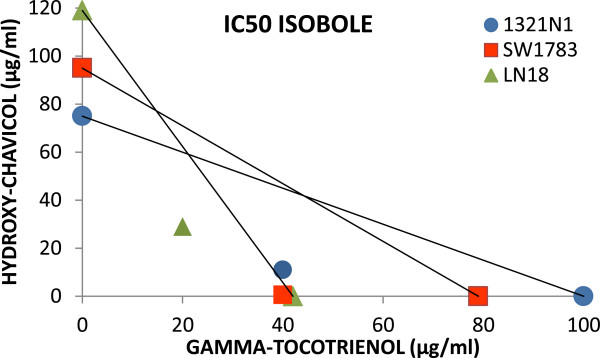
**Isobologram analysis of GTT and HC antiproliferative effects on glioma cells.** Individual IC50 doses for GTT and HC were calculated and then plotted on the x and y axes. The line connecting these points represents the drug doses of each compound that would induce the same growth inhibition when used in combination if the interaction between these compounds were additive. The data point on the isobologram represents the actual doses of combined GTT and HC treatment that results in 50% growth inhibition. Since the data point is positioned well below the line, a synergistic anti-proliferative effect is indicated.

The IC50 data obtained were plotted for analysis of synergism by isobole diagram (Figure 3) and the combination index (CI) was obtained. Analysis of the isobologram and the interaction values revealed that all the bioactive combinations showed synergism. Combined sub-effective doses of GTT + HC enhanced the inhibition of glioma cell proliferation and exhibited a strong synergistic effect on 1321N1 and SW1783 with CI values of 0.55 and 0.54 respectively. In LN18 cells, moderate synergism interaction of GTT + HC was observed with CI = 0.73 (Table 2).

### The effect of combined GTT + HC bioactives on apoptosis

**Figure 4:**
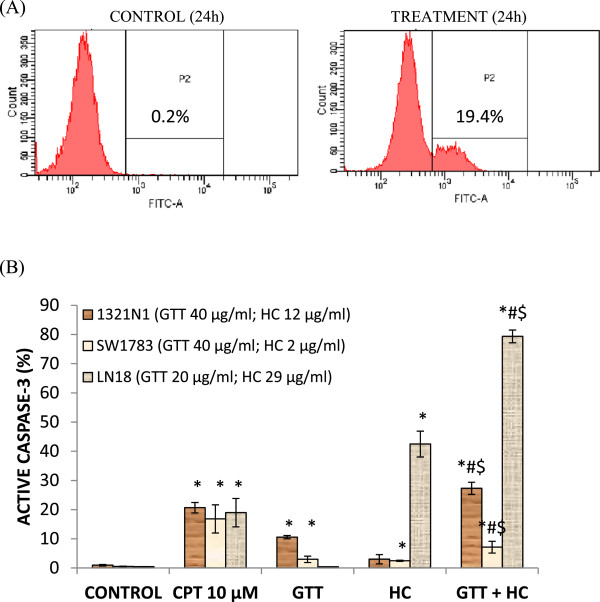
**Detection of apoptosis using flow cytometry after caspase-3 antibody staining.** 1321N1, SW1783 and LN18 cells were treated with combined treatments at IC50 concentrations for 24 h. **(A)** An example of active caspase-3 detection diagram from 1321N1 cells: viable cells are in the left quadrant, while the presence of active caspase-3 is shown in right quadrant (P2). **(B)** Combined compounds caused greater inhibition of growth of 1321N1, SW1783 and LN18 cells than either agent alone as evidence by the presence of active caspase-3. Each value represents mean ± SD of three independent experiments. *P < 0.05 compare to control. # *P* < 0.05 compare to GTT. $*P* < 0.05 compare to HC.

**Figure 5:**
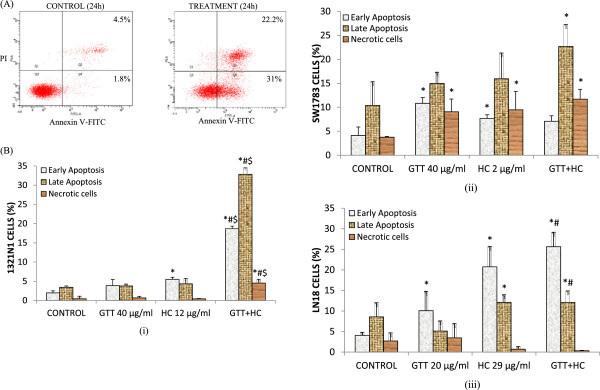
**Combined treatments potentiate apoptosis mediated cell death in 1321N1, SW1783 and LN18 cells. (A)** Detection of apoptosis using flow cytometry after annexin V-FITC/propidium iodide (PI) staining. 1321N1, SW1783 and LN18 cells were treated with combined treatments at IC50 concentrations for 24 h. Viable cells are in the lower left quadrant (Q3), early apoptotic cells are in the lower right quadrant (Q4), late apoptotic cells are in the upper right quadrant and non-viable necrotic cells are in the upper left quadrant (Q1). **(B)** Bar graph representing mean values from three independent experiments for i.1321N1, ii. SW1783 and iii. LN18. *P < 0.05 compare to control early or late apoptosis respectively. # *P* < 0.05 compare to GTT early or late apoptosis respectively. $*P* < 0.05 compare to HC early or late apoptosis respectively.

Characteristics of 1321N1, SW1783 and LN18 glioma cells undergoing apoptosis while treated with combined bioactives were determined by the presence of active caspase-3 and the staining of exposed phosphatidylserine on the cell surface which is demonstrated during annexin-V FITC/PI flow cytometry assay. A set of results from Active Caspase-3 Apoptosis assay and Annexin-V Apoptosis assay were illustrated in Figures 4 and 5 respectively. Treatment of combined GTT + HC on 1321N1 resulted in 27.3% activation of caspase-3 compared to either GTT (10.5%) or HC (3%) alone. There was only a slight increase of active caspase-3 (7.1%) in SW1783 when treated with combined GTT + HC compared to individual compounds GTT (3%) or HC (2.5%). However, combined GTT + HC treatment on LN18 resulted in 79.4% activation of caspase-3 compared to either GTT (0.5%) or HC (42.5%) alone (Figure 4). Highest induction (79.4%) of active caspase-3 was observed in LN18 when treated with combination compounds compared to both 1321N1 and SW1783.Similar results were obtained for double fluorescence staining of annexin-V FITC/PI flow cytometry assay. As shown in Figure 5B(i), the percentage of both early (18.7%) and late apoptotic cells (32.8%) for GTT + HC treatment in 1321N1 cells increased significantly compared to GTT or HC treatment alone. Early apoptosis (25.6%) was increased in LN18 treated with combined GTT + HC when compared to GTT or HC monotherapy respectively (Figure 5B(iii)). However, no significant changes were seen in the percentage of late apoptosis for LN18 cells treated with GTT + HC compared to HC alone. In contrast, SW1783 revealed increased late apoptotic cells (22.7%) with treatment of combined GTT + HC, while early apoptotic cells were found to be decreased with combined GTT + HC treatment (7.1%) compared to GTT alone (10.8%) (Figure 5B(ii)). Interestingly, the percentage of necrotic or dead cells were found to be elevated in SW1783 treated with GTT + HC (11.7%), as well as GTT (9.1%) or HC (9.5%) (Figure 5B(ii)) compared to necrotic cells in 1321N1 (Figure 5B(i)) and LN18 (Figure 5B(iii)) cells which were below than 5%.

### The effect of combined GTT + HC on normal cells

**Figure 6:**
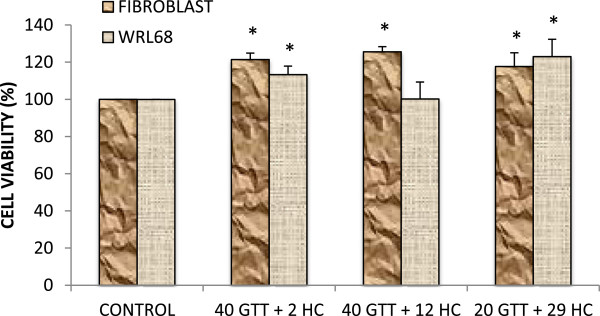
**Bar graph of normal foreskin fibroblast and normal liver WRL68 cells treated with combined GTT + HC after 24 hours.** No sign of toxicity were observed. Data represent the mean ± SD (n = 3).

Treatment of combined GTT + HC using IC50 doses obtained from the MTS assay data on normal embryonic liver cells (WRL 68) and normal foreskin fibroblast cells showed no signs of toxicity. Moreover, most of the treatment doses of GTT + HC tested significantly increased cell proliferation of these cells (Figure 6).

## Discussion

Recently, there is increased interest in the search for potential chemopreventive agents for treatment against various types of cancer particularly as adjuvant therapy and in the prevention of recurrence. Insights on the mechanism of natural bioactives in regulating cell survival and proliferation are important in the development of new agents that can be utilized in low toxicity without the severe side effects accompanying chemotherapy. The present study provides evidence that both GTT and HC act as a potent growth inhibitory compound in human glioma cancer cells. Our study suggests that LN18 (grade IV) cells were more sensitive to GTT treatment alone with an IC50 (42 μg/ml) value which is much lower than grade II 1321N1 (100 μg/ml) and grade III SW1783 cells (79 μg/ml). As for HC treatment, 1321N1 (grade II) cells were more susceptible to HC alone with IC50 (75 μg/ml) much lower than grade III SW1783 (95 μg/ml) and grade IV LN18 cells (119 μg/ml) (Table 1). Although further studies are required, these low concentrations maybe achievable in terms of plasma concentrations suggesting achievable bioavailability of these natural bioactives. These early findings also suggested that different grade tumours responded differently to the bioactives and possibly target different sites of action.

In a number of cell types, GTT or HC were found to produce an anticancer activity and induce apoptosis. Tocotrienols has anti-angiogenic properties [7], while HC was reported to modulate immune responses [11]. Previous investigation have shown that GTT induces growth arrest through several pathways such as through the suppression of ß-catenin/Tcf signaling in human colon cancer HT29 cells [21] and has been reported to modulate a novel pathway through down-regulation of TGFß2 in prostate cancer [22]. Furthermore, studies by S Shah, A Gapor and PW Sylvester [23] and S Shah and PW Sylvester [24] revealed that GTT induced apoptosis by caspase-8 and caspase-3 but not caspase-9 activation in neoplastic mammary epithelial cells. HC was shown to possess antibacterial activity at high concentration of 500 μg/ml and inhibited MCF-7 cell growth at IC50 of 200 μM [25]. Each of the glioma cell lines tested in this study possess different characteristics. For example, 1321N1 cells contain M2-gland muscarinic receptors [26], while some of the characteristics of SW1783 cell lines include the presence of PDGFRA (4q11) amplification, wild type CDKN2A, PIK3CA (3q26.3) one copy loss and low-level copy number gain of BIRC5 (17q25) [27]. While LN18 was identified to carry a p53+ mutated gene, PTEN + wild type, p16- deleted and p14ARF- deleted gene, besides has been reported to be negative for glial fibrillary acidic proteins and S-100 proteins [28-30]. Perhaps, specific characteristics such as different mutations involved in different grades of glioma cell lines influenced the choice of route of signaling pathway mediated by GTT and HC in order to inhibit the proliferation of glioma cells [31]. This is further supported by evidence which have shown that tocotrienols mediated its apoptotic effect through activation of different intracellular signaling mechanisms in different types of cancer cells [8]. This may provide an explanation for the different doses of each bioactives required to inhibit proliferation of the cancer cells in culture [31].

Since multiple signal transduction pathways become dysregulated in glioma cancer, an improvement in inhibition of tumor growth could be achieved with combination therapies that affect several targets and possibly override different drug resistance [1,32]. This is consistent with the observation that the effectiveness of anticancer chemotherapy may be improved when multiple drugs with complimentary mechanisms of action are used in combination. Combination therapy optimizes the effectiveness of each treatment by their complimentary action that will eventually result in synergistic therapeutic response, while at the same time reducing toxic adverse side effects associated with high dose monotherapy [5,15]. Evidence have shown that combined low dose tocotrienol treatment with specific chemotherapeutic drugs such as statins [33] and celecoxib [34] showed significantly enhanced therapeutic response compared to that observed from individual treatments alone. It is possible that combination of GTT + HC is more effective than narrowly focused therapies as each bioactive are likely to impact several aspects of tumor progression. Our results further confirmed and extended the findings from previous studies where a synergistic anti-proliferative response were observed in grade II, III and IV human glioma cell lines after exposure to lower dose combination treatment with GTT and HC compared to individual treatment alone (Table 2).

The reduction in cell proliferation and increased apoptosis are regarded as one of the key strategies for cancer prevention. As cell death could be divided into necrosis or apoptosis, it is essential to determine the mode of cell death induced by combined GTT + HC treatment. Apoptosis is a programmed cell death and unlike necrosis, does not trigger inflammatory responses [35]. SW1783 cells were observed to finally progress to secondary necrosis (Figure 5B(ii)). It should be emphasized that secondary necrosis differs from normal necrosis. During apoptotic event *in vivo*, the presence of macrophage or neighboring cells facilitated the disposal of apoptotic bodies; thereby avoiding lysis. However, due to the absence of phagocytes, the apoptotic bodies produced *in vitro* will eventually swell and lyse [36]. As GTT, HC and their combination induced cell death follow the sequence of early apoptosis, late apoptosis and secondary necrosis it is concluded than the treatment actually induce cell death via apoptotic rather than necrotic pathway.

Synergism between the GTT and HC bioactives may be due to the fact that the bioactives acting in concert to reach different targets of the same signalling pathway, controlling apoptosis, thus accelerating cell death process. The effectiveness of well-chosen combinations has been proven by a study on prostate cancer [37] where the combinations of EGCG, genistein and quercetin trigger apoptosis in CWR22R*v*1 via multiple actions, not through direct inhibition of the tumor but more precisely, by suppression or activation of different processes, which are critical for the tumor’s survival. Consistent with our *in vitro* data, sub-effective doses of GTT + HC induce greater effect in glioma cells compared to GTT or HC compound alone (Figure 4B and Figure 5B).

The result of combined GTT + HC treatment are similar in causing cell death, but perhaps, due to the different mutations in 1321N1, SW1783 and LN18, the modulation of apoptosis by GTT + HC may be through different pathways. This was observed in a study by Zhang et al. [38] where the variation in induction of apoptosis by combined all*-trans* retinoic acid (ATRA) and/ or interferon gamma (IFN-γ) might be attributed to the difference in PTEN expression in LN18 (PTEN-proficient) and U87MG (PTEN-deficient) cells. Moreover, gene expression profiling in estrogen receptor positive, p53 wild-type MCF-7 and estrogen receptor-negative, p53 mutant MDA-MB 231 cells treated with tocotrienol-rich fraction of palm oil suggested different mechanisms in the two cell lines [9].

Vitamin E is a well known antioxidant which scavenges free radicals and may play a role in preventing oxidative damage to the prostate epithelium. However, the ability of GTT, an isomer of vitamin E, in inducing apoptosis in cancer cells may not necessarily correlate to its antioxidant characteristics, but GTT may modulate gene-regulatory functions through mechanisms unrelated to their antioxidant properties. For example, GTT down-regulates cyclin D1 and cyclin E levels in several cancer cell lines. Furthermore, targeting the mevalonate pathway by tocotrienols for cancer therapy and prevention is currently under investigation [22].

Evidence suggests that HC elicit cytotoxicity through mitochondrial failure related to mitochondrial membrane potential at an early stage and subsequently lipid peroxidation through oxidative stress at a later stage [39]. This causes release of cytochrome c from mitochondria, cleavage of caspase 9, 3 and poly-adenosine diphosphate-ribose polymerase (PARP) leading to apoptosis. Interestingly, HC was recently found to induce apoptosis of leukemic (CML) cell lines expressing mutated Bcr-Abl with imatinib resistance phenotype [12]. HC also showed scavenging properties toward H_2_O_2_, superoxide and hydroxyl radicals and is possibly an antioxidant at low concentrations whereas at higher concentrations, HC induces glutathione (GSH) depletion, reactive oxygen species (ROS) production, cell cycle arrest and apoptosis of oral KB epithelial cells (cell death mediated by oxidative stress) [10]. A study by Chen et al. [40] tested the potential cytotoxic effects of HC in metabolically competent human liver derived HepG2cells by examining the modulation factors that may contribute to HC-induced toxicity and apoptotic effects, and the relationship to oxidative stress [40].

Furthermore, both tocotrienols [41] and HC [11] have been reported to be capable of crossing the blood–brain barrier. Therefore, the use of combined GTT + HC is an attractive approach for cancer chemoprevention in glioma cancer due to the bioavailability of these bioactives in the brain. Finally, GTT displays potent anticancer activity against numerous cancer cells at treatment doses that have little or no effect on normal cell function and viability [7], while HC was shown to induce cell death of leukemic cell lines with minimal toxicity to normal peripheral blood mononuclear cells [12]. Combined GTT + HC may be considered as potential therapy for glioma cancer treatment as it is able to selectively induce apoptosis in grade II, III and IV cells in this study without affecting normal embryonic liver cells (WRL 68) and normal foreskin fibroblast cells (Figure 6), while most of the treatment doses of GTT + HC tested increased cell proliferation of these normal cells.

Overall, synergy effects in pharmacology area are already known, but their exact mechanisms have yet to be elucidated. Although tocotrienols have the potential to be chemotherapeutic or preventive agents in the human diet and HC has been reported to possess anticancer activity, their exact mechanisms of action on cell death and other inhibitory pathways are less known. Future studies on the elucidation of mechanisms induced by combined GTT + HC are warranted.

## Conclusions

Data from our *in vitro* studies demonstrated that combined GTT + HC treatment synergistically induced anticancer response mediated by apoptosis via caspase-3 in 1321N1, SW1783 and LN18 than either bioactives alone. Furthermore, different ratios of combined GTT + HC were needed to achieve the enhanced inhibitory effect against different grades of glioma cell lines.

## Competing interests

The authors declare that there are no competing interests.

## Authors’ contributions

AAR: performed experiments, analysis and interpretation of data and wrote the manuscript. ARAJ, RH and NMM: involved in general supervision. WZWN: designed the study, revised the manuscript and supervised this study. All authors have read and approved the manuscript.

## Pre-publication history

The pre-publication history for this paper can be accessed here:

http://www.biomedcentral.com/1472-6882/14/213/prepub
